# Measurement of the reaction enthalpy of CO_2_ in aqueous solutions with thermographic and gravimetric methods

**DOI:** 10.1038/s41598-024-61242-9

**Published:** 2024-05-06

**Authors:** Jessica Jung-Fittkau, Josef Diebold, Andrea Kruse, Hans-Peter Deigner, Magnus S. Schmidt

**Affiliations:** 1 Organic and Bioorganic Chemistry Labs, Medical and Life Sciences Faculty, Institute of Precision Medicine, Furtwangen University, Jakob-Kienzle-Str. 17, 78054 Villingen-Schwenningen, Germany; 2 Institute of Agricultural Technology, University of Hohenheim, Schloß Hohenheim 1, 70599 Stuttgart, Germany; 3 EXIM Department, Fraunhofer Institute IZI (Leipzig), Schillingallee 68, 18057 Rostock, Germany; 4 Faculty of Science, Eberhard Karls University Tuebingen, Auf der Morgenstelle 8, 72076 Tübingen, Germany

**Keywords:** Thermography, Gravimetry, Reaction enthalpy, MEA, CO_2_ capturing, Analytical chemistry, Organic chemistry, Physical chemistry, Environmental sciences

## Abstract

In this work, a new concept for the approximate determination of the reaction enthalpy of the reaction between CO_2_ and monoethanolamine (MEA) in aqueous solution was developed. For this purpose, a CO_2_ gas stream was flowed into aqueous MEA solutions with different concentrations of 1 wt%, 2.5 wt% and 7.5 wt%. The weight difference ∆T, which is based on the increase in CO_2_ bound by the MEA over time, was documented using a thermographic camera. The mass difference ∆m, which is also based on the increase in CO_2_ bound by the MEA over time, was determined using a balance. By determining ∆T and ∆m, an approximate calculation of the reaction enthalpy is possible. The deviation from the values from the data known from the literature was less than 5% in all experiments.

## Introduction

The amount of anthropogenic greenhouse gas emissions, especially the concentration of CO_2_, in the atmosphere is steadily increasing since the industrial revolution in the nineteenth century and there is no sign of this trend reversing^1–3^. The greenhouse gases the mankind emits has serious consequences for our planet, such as global warming. If the earth warms too much, it can negatively influence all life on earth^3,4^.

An important goal to the present and future generations should be to find solutions to reduce greenhouse gas emissions. The solution to this problem is not just to reduce the greenhouse gas emissions alone^5^. A very promising approach for CO_2_ capture and storage is gas scrubbing with an aqueous solution containing ethanolamine^6–9^. Gas scrubbing enables end-of-the-pipe technology, which means that existing industrial plants can be retrofitted with such technology and do not need to be replaced^5,10,11^. The absorption of acid gases in alkaline solutions is an already established industrial process for the treatment of gases^1,12,13^.

When CO_2_ is bound in an aqueous MEA solution, the CO_2_ is first dissolved in the water and then binds to the amine (cf. Eq. 1)^1^.1

This chemical reaction releases energy in the shape of heat. It is known that the reaction enthalpy is approx. − 854 kJ mol^-1^.^14,15^.

In general, the calculation of the reaction enthalpy is described by the Eq. 2^16^:

2 $$ \Delta HR \, = \, \Delta U \, + \, p\Delta V $$

In Eq. 2, ∆U is the energy difference between the initial state and the final state of the system and p∆V is the volume work. The enthalpy change in a chemical reaction is expressed by the reaction enthalpy. The reaction enthalpy indicates whether a chemical reaction is endothermic or exothermic. If energy is released during the reaction, the reaction is exothermic; if energy is required, it is endothermic. For exothermic reactions, ∆HR < 0 (energy is released) and for endothermic reactions ∆HR > 0 (energy is absorbed by the system)^16–18^.

The reaction enthalpy plays an important role in the scale-up of chemical processes and in calculations related to process safety^19^. Until now, the reaction enthalpy of chemical reactions has been determined using calorimetry. There is direct and indirect calorimetry. In direct calorimetry, the heat quantities are determined using a calorimeter. Your calorimeter is an insulated apparatus in which the reaction can take place. The heat that is released or required for the reaction is measured. In indirect calorimetry, the amount of heat released is calculated indirectly via the measured substance consumption^20,21^.

Thermography is a process in which temperature changes can be measured. In this work, thermography was used to track chemical reactions. Chemical reactions are either endothermic or exothermic. In an endothermic reaction, energy is used so that a decrease in temperature can be observed. In an exothermic reaction, energy is released so that an increase in temperature can be observed. These temperature differences can be measured with a thermographic camera^22^.

This article describes a method for the approximate determination of the reaction enthalpy of chemical reactions in aqueous phase with a gas using the reaction of CO_2_ with MEA. In contrast to classical calorimetry, this new method does not require a closed system. It offers a quick and easy way to determine reaction enthalpies approximately without the use of a calorimeter. The reaction of MEA with CO_2_ in water was chosen because the enthalpy of reaction is already known in literature and is therefore suitable for evaluating the new method. The experiments were carried out with aqueous MEA solutions of different concentrations at room temperature.

## Material and methods

### Calculation method of the reaction enthalpy

The mass differences determined in the gravimetric experiments were used to calculate the molar quantity of CO_2_ bound in the MEA solution.

By definition, it takes 1 J to heat 1 g of water by 0.239 K^23^. This means that it is possible to deduce the energy released during the chemical reaction from the temperature differences determined by the thermographic camera.

By dividing the temperature difference determined in the thermographic experiments by 0.239 K g J^−1^, the energy released per g of solution during the reaction could be calculated (specific energy e [J g^−1^]). The aqueous MEA solution was assumed to have the same properties as water due to its dilution (cf. Eq. 3).

3 $$e [{{\text{Jg}}}^{-1}] =\frac{\Delta T\left[{\text{K}}\right]}{\mathrm{0,239} \left[{{\text{KgJ}}}^{-1}\right]}$$

This value was then multiplied by the mass of solution used and divided by the molar quantity of gas absorbed, resulting the reaction enthalpy of the reaction (cf. Eq. 4).

4 $$\Delta {H}_{R} \left[{{\text{Jmol}}}^{-1}\right]=-\frac{e \left[{{\text{Jg}}}^{-1}\right]*m [{\text{g}}]}{n \left[{\text{mol}}\right]}$$

### Gravimetric experiments

**Figure 1:**
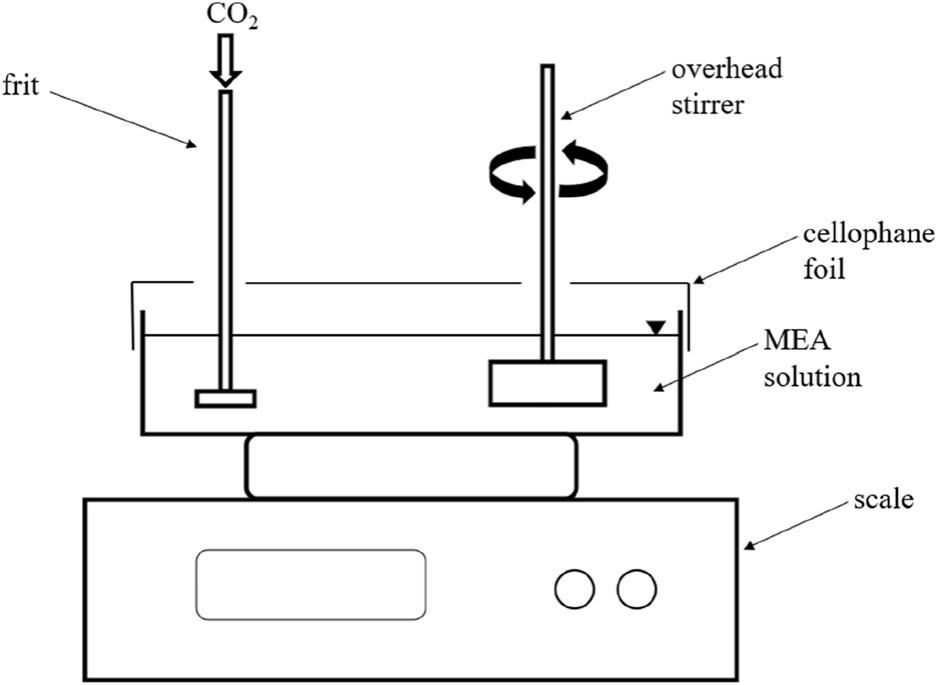
Experimental setup of the gravimetric experiments.

The gravimetric experiments were carried out in a vessel measuring 20 × 10 × 6 cm with different MEA solutions (cf. Fig. 1). The mass of the solutions used for each experiment was 200 g. The solution was aerated with 40 l h^−1^ CO_2_ via a glass frit. Stirring was performed via a overhead stirrer with 250 rpm. A cling film was used as a splash guard, which had openings for inserting the frit and the overhead stirrer. The experimental setup was placed on a precision balance. The weight was recorded once per minute. Each experiment was conducted three times.

### Thermographic experiments

**Figure 2:**
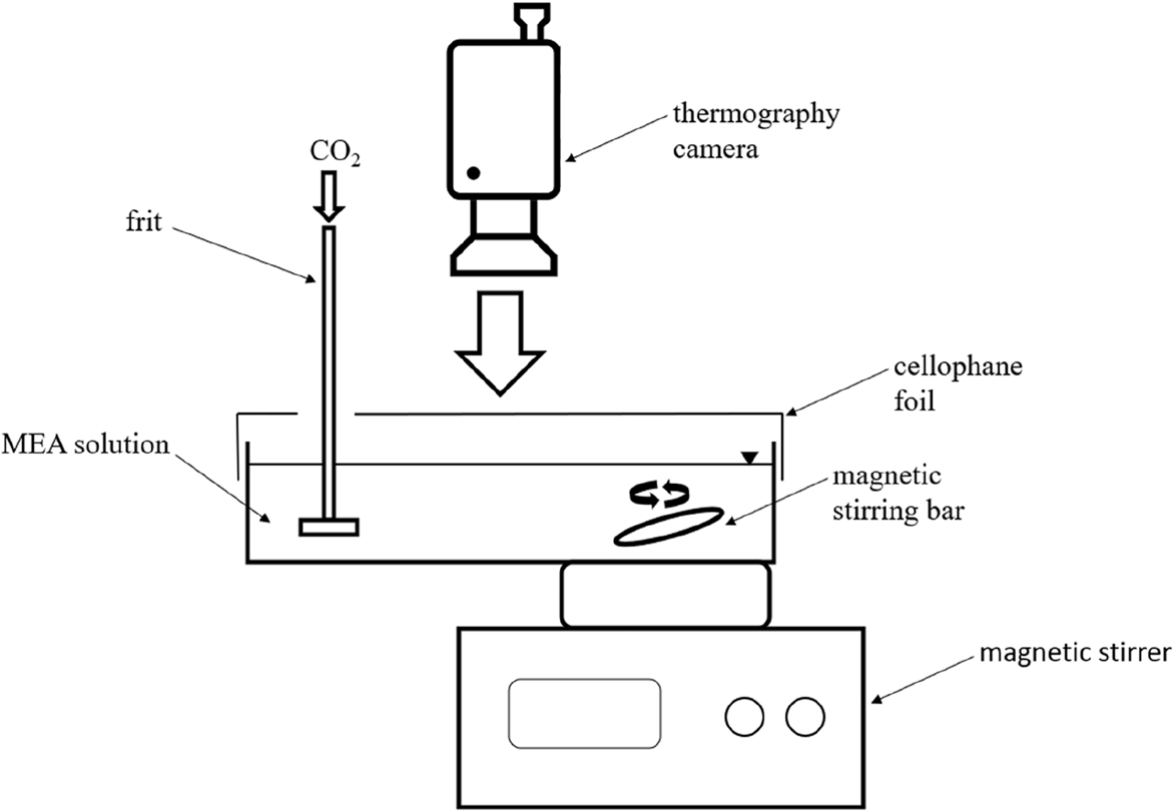
Experimental setup of the thermographic experiments.

The thermographic experiments were carried out in a vessel measuring 20 × 10 × 6 cm with different MEA solutions (cf. Fig. 2). The mass of the solutions used for each experiment was 200 g. The solution was aerated with 40 l h^-1^ CO_2_ via a frit. The mixing was done via a magnetic stirrer with 250 rpm. A cling film was used as a splash guard, which had openings for inserting the frit. The thermographic camera (VarioCAM HD head 980 S by the company InfraTec) monitored the reaction vertically from the top. The magnetic stirrer and the camera were warm-up for 30 min so that no falsification of the data could take place through the electrics. The evaluation of the data was done by the software IRBS 3.1. Each experiment was conducted three times.

## Results and discussion

### Results and discussion of the gravimetric experiments

The mass difference (∆m) was used in the evaluation of the gravimetric data, as the mass changing over time was measured in the gravimetric tests with the balance (Supplementary information 1).

**Figure 3:**
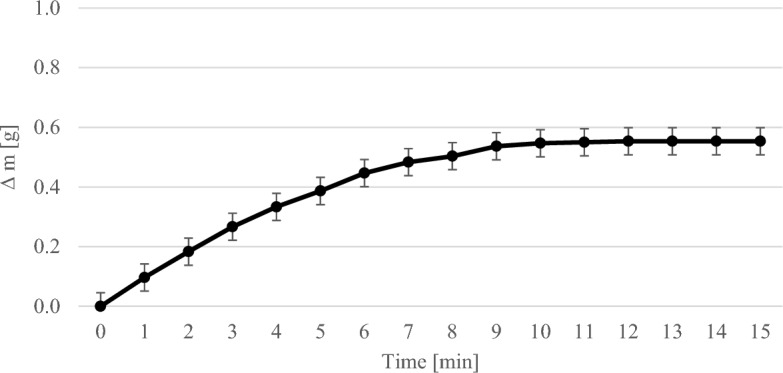
Results of the gravimetric experiments with 1 wt% MEA solution.

Figure 3 shows the course of the gravimetric experiments with 1 wt% MEA. The error bars show the standard deviation of the repetitions of the experiment. The increase in mass was plotted against time. After about 10 min the MEA solution became saturated.

**Figure 4:**
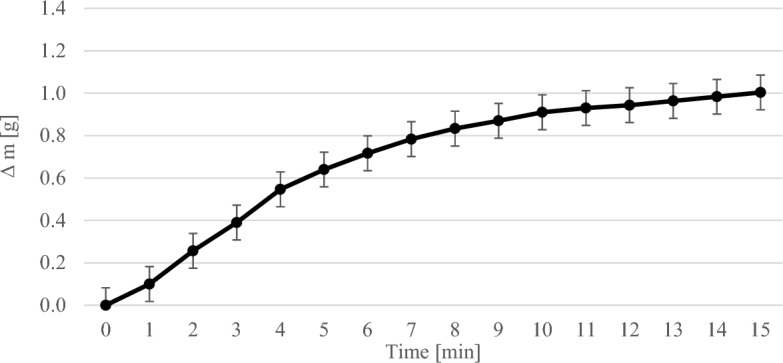
Results of the gravimetric experiments with 2.5 wt% MEA solution.

Figure 4 shows the course of the gravimetric experiments with 2.5 wt% MEA. The error bars show the standard deviation of the repetitions of the experiment. The weighted mass difference was plotted against time. As one can see that the maximum amount of CO_2_ which can be absorbed by the 2.5 wt% MEA solution was not yet reached after 15 min.

**Figure 5:**
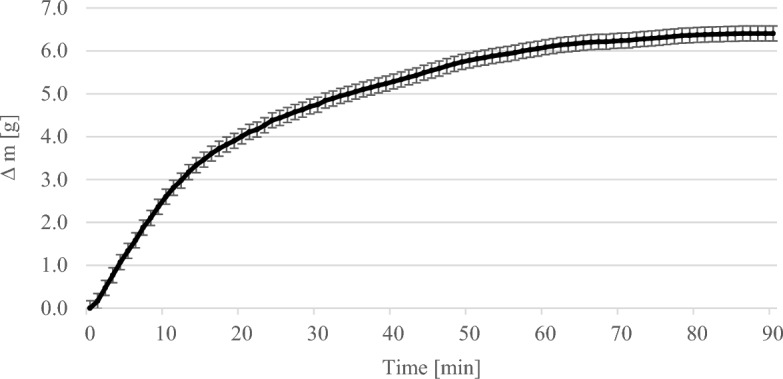
Results of the gravimetric experiments with 7.5 wt% MEA solution.

Figure 5 shows the course of the gravimetric experiments with 7.5 wt% MEA. The error bars show the standard deviation of the repetitions of the experiment. The weighed mass difference was plotted against time. It can be seen that the MEA solution became saturated at around 80 min and the weight reached its maximum.

In general, the mass of the solution increased steadily as more and more CO_2_ was bound by the MEA solution over time until saturation occurred. The higher the concentration of MEA in the solution, the more CO_2_ could be bound. At a MEA concentration of 1 wt% it was 0.55 g per 200 ml solution, at 2.5 wt% 1 g per 200 ml solution and at 7.5 wt% 6.41 g per 200 ml solution. The more MEA there was in the solution, the longer it took for saturation to occur. At 1 wt% MEA the time was about 10 min and at 7.5 wt% about 80 min.

In order to assess these findings, gravimetric experiments were also carried out with water instead of the aqueous MEA solution. Here, no change in weight was observed over time.

Possible sources of error in the gravimetric experiments can be fluctuations in the gas volume flow, which exert pressure on the balance and thus falsify ∆m. Temperature fluctuations, which influence the rection of CO_2_ with MEA, could also be a source of error.

### Results and discussion of the thermographic experiments

**Figure 6:**
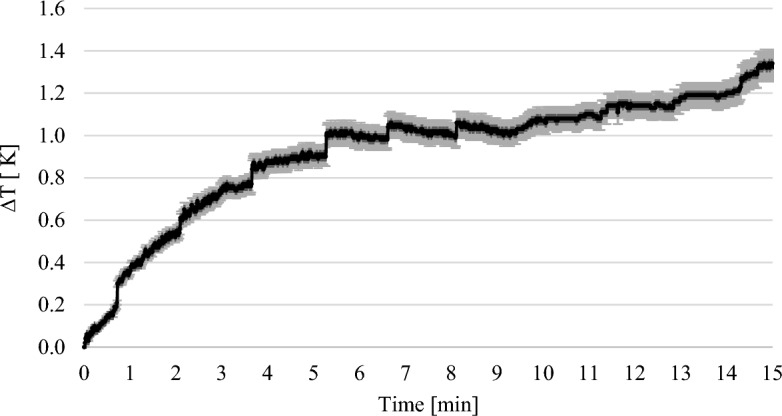
Results of the thermographic experiments with 1 wt.% MEA solution.

Figure 6 shows the course of the thermographic experiments with 1 wt% MEA. The error bars show the standard deviation of the repetitions of the experiment. The weighted temperature difference was plotted against time. This graph shows that the maximum temperature was not reached even after 15 min.

**Figure 7:**
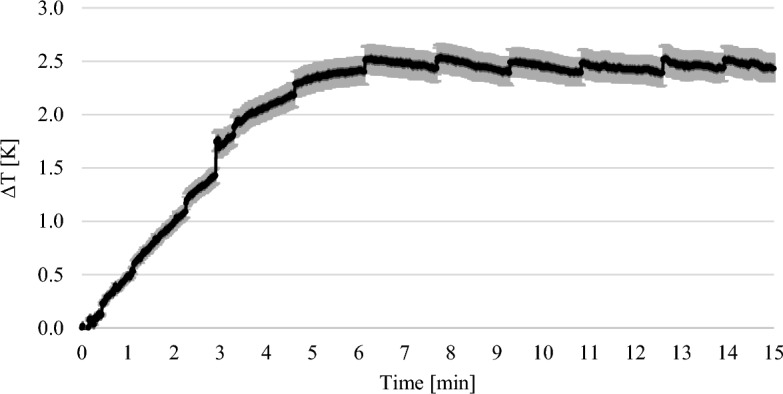
Results of the thermographic experiments with 2.5 wt% MEA solution.

Figure 7 shows the course of the thermographic experiments with 2.5 wt% MEA. The error bars show the standard deviation of the repetitions of the experiment. The weighted temperature difference was plotted against time. In contrast to the result at 1 wt% the maximum temperature was reached after approximately 6 min.

**Figure 8:**
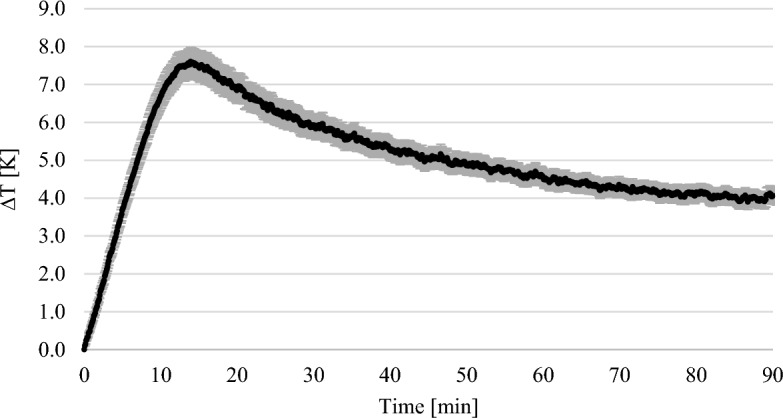
Results of the thermographic experiments with 7.5 wt% MEA solution.

Figure 8 shows the course of the thermographic experiments with 7.5 wt% MEA. The error bars show the standard deviation of the repetitions of the experiment. The weighted temperature difference was plotted against time. The graph shows that the maximum temperature was reached after about 13 min. The temperature then dropped again.

The weight of the solutions increased steadily in the gravimetric experiments until a maximum was reached (see Figs. 3, 4, 5). In the thermographic experiments, the temperatures also increased initially until a maximum was reached. However, this maximum was always at an earlier point in time than in the gravimetric experiments. In the experiments with 7.5 wt% MEA solution, for example, the gravimetric maximum was reached after about 80 min (cf. Fig. 5), the temperature maximum after about 13 min (cf. Fig. 8). The temperature then dropped again. After 13 min, the reaction in the thermographic experiments continued in exactly the same way as in the gravimetric experiments, but obviously the reaction rate became less which leads to the drop of temperature.

In this work, thermographic experiments were also carried out with water instead of the aqueous MEA solution. No change in temperature was observed over time.

Possible sources of error in the thermographic experiments can be strong temperature fluctuations in the environment, which on the one hand influence the reaction of CO_2_ with MEA and, on the other hand, falsify the complete thermographic measurement (Supplementary information 1).

### Results and discussion of the calculation of the reaction enthalpy

The temperature maxima from the thermographic experiments and the corresponding data from the gravimetric experiments at the same time were used to calculate the enthalpy (Supplementary information 1).

In the experiments with 1 wt% MEA, ∆T was used at a time of 15 min. At this time, ∆T was 1.34 K (cf. Fig. 6). The ∆m of the solution in the gravimetric tests was 0.55 g after 15 min (cf. Fig. 3). Thus, the calculated enthalpy was − 89.21 kJ mol^−1^ and deviates from the literature values by 4.46%^14^.

In the experiments with 2.5 wt% MEA, ∆T was used at the time of 13–15 min. ∆T at this time was 2.44 K (cf. Fig. 7). The ∆m of the solution in the gravimetric experiments was 1 g after 15 min (cf. Fig. 4). Thus, the calculated enthalpy was − 89.56 kJ mol^-1^ and deviats from the literature values by 4.87%^14^.

In the experiments with 7.5 wt% MEA, ∆T was used at the time of 14 min. ∆T at this time was 7.6 K (cf. Figure 8). The ∆m of the solution in the gravimetric experiments was 3.34 g after 14 min (cf. Figure 5). Thus, the calculated enthalpy was − 83.88 kJ mol^-1^ and deviates from the literature values by 1.78%^14^.

Overall, the deviation of the enthalpies determined according to the described method was less than 5% in all experiments^14^.

Possible sources of error in the calculation of the reaction enthalpy could be that the temperature maximum of the reaction is determined incorrectly and the wrong mass is used for the calculation at the wrong time.

## Outlook and conclusion

Using the reaction of CO_2_ in an aqueous MEA solution as an example, a new concept for the approximate determination of the enthalpy of the reaction of a gas in an aqueous solution was successfully developed in this work. For this purpose, a CO_2_ gas flow of 40 L h^−1^ was fed into aqueous MEA solutions of various concentrations and ∆T was determined with the aid of a thermographic camera and ∆m with a balance. MEA solutions with a concentration of 1 wt%, 2.5 wt% and 7.5 wt% were used for this purpose. In the experiments with 1 wt% MEA, the enthalpy determined was − 89.21 kJ mol^−1^, in the experiments with 2.5 wt% MEA -89.56 kJ mol^−1^ and in the experiments with 7.5 wt% MEA − 83.88 kJ mol^−1^. The enthalpy stated in the literature is − 85.4 kJ mol^−1^^14^. The deviation from the values in the literature was therefore less than 5% in all experiments. The reaction enthalpy plays an important role in the scale-up of chemical processes and in calculations related to process safety^19^. With the method developed in this work, it is possible to fast and simple determine the reaction enthalpy of reactions of gases in an aqueous solution.

### Supplementary Information

Supplementary Information.

## Data Availability

The datasets generated during and/or analyzed during the current study are available from the corresponding author on reasonable request.
